# Supplementary material for: Creation of a Biobank of the Sperm of the Honey Bee Drones of Different Subspecies of Apis mellifera L
Source: Animals (Basel). 2023 Nov 28;13(23):3684. doi: 10.3390/ani13233684 (PMC10705684; doi:10.3390/ani13233684)
Supplement: Supplementary file 1 [file animals-13-03684-s001.zip › Figure S1.pdf]

Figure S1 The studied morphometric parameters

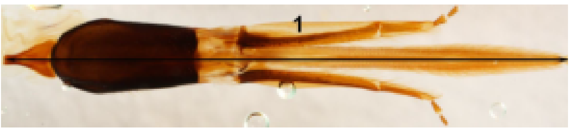

(1) proboscis length *Lx*

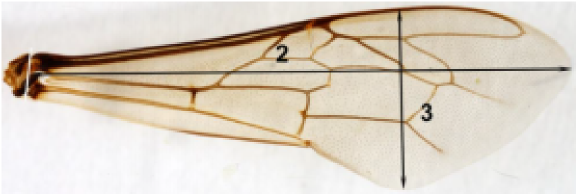

(2) fore wing length F_L and (3) width F_W

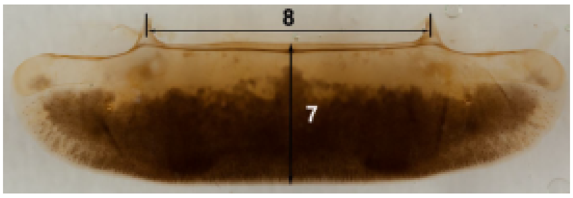

(7) length *Lt3* and (8) width *Wt3* of third tergite

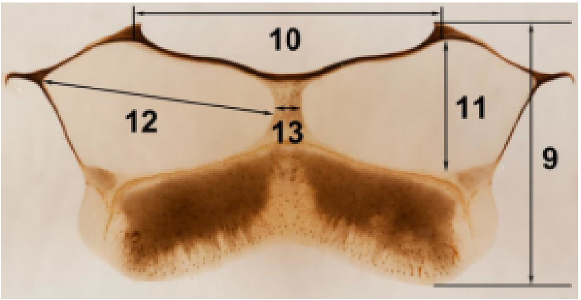

(9) length of third sternite *Ls3*, (10) width of third sternite (this parameter was not used), (11) length *Lwm* and (12) width *Wwm* of wax mirror, (13) distance between wax mirrors *Lwmd*

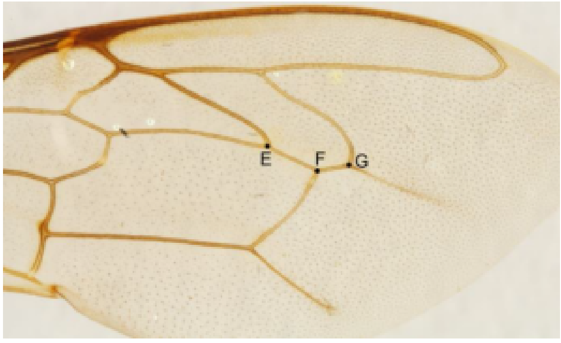

Linear dimensions EF and FG for CI assessment (EF/FG*100%=CI)

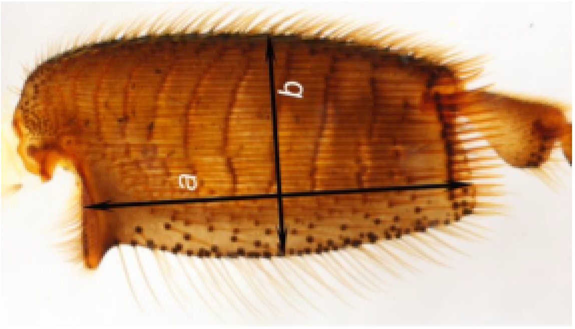

Linear dimensions *a* (length) and *b* (width) on the first tarsal segment of the right hind leg for TI assessment (b/a*100%=TI)
