# Supplementary figures and images for: Creation of a Biobank of the Sperm of the Honey Bee Drones of Different Subspecies of Apis mellifera L
Source: Animals (Basel). 2023 Nov 28;13(23):3684. doi: 10.3390/ani13233684 (PMC10705684; doi:10.3390/ani13233684)

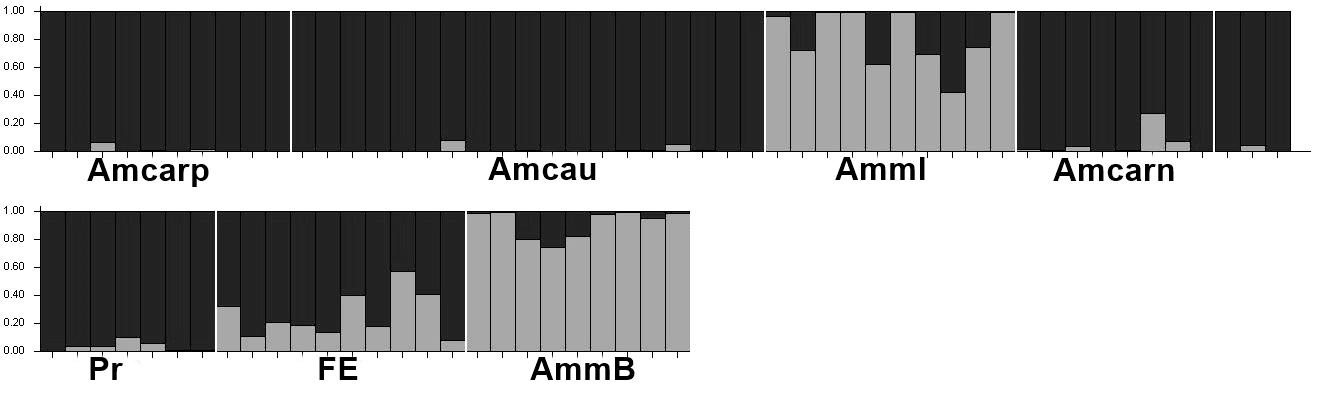

Supplement: Supplementary file 1 [file animals-13-03684-s001.zip › Figure S2.JPG]
